# Synthesis, crystal structure, and Hirshfeld surface analysis of undeca­carbon­yl{tris­[4-(methyl­sulfan­yl)phen­yl]arsine}-*triangulo*-triosmium(0)

**DOI:** 10.1107/S2056989025006541

**Published:** 2025-07-29

**Authors:** Siti Syaida Sirat, Mohd Mustaqim Rosli, Sang Loon Tan, Ibrahim Abdul Razak, Omar bin Shawkataly

**Affiliations:** a Faculty of Applied Sciences, Universiti Teknologi MARA, Cawangan Negeri Sembilan, Kampus Kuala Pilah, 72000 Kuala Pilah, Negeri Sembilan, Malaysia; b Atta-ur-Rahman Institute for Natural Product Discovery (AuRIns), Universiti Teknologi MARA, Kampus Puncak Alam, 42300 Bandar Puncak Alam, Selangor, Malaysia; c X-ray Crystallography Unit, School of Physics, Universiti Sains Malaysia, 11800 USM, Penang, Malaysia; d Sunway Biofunctional Molecules Discovery Centre, Faculty of Medical and Life Sciences, Sunway University, 47500 Sunway City, Malaysia; e Department of Chemistry, Faculty of Science, Universiti Teknologi Malaysia, 81310 Skudai, Johor Bahru, Malaysia; University of Neuchâtel, Switzerland

**Keywords:** osmium, crystal-structure, cluster, Hirshfeld surface analysis

## Abstract

The title compound, [Os_3_(C_21_H_21_AsS_3_)(CO)_11_] or [Os_3_{As(C_6_H_4_SCH_3_)_3_}(CO)_11_], comprises a triangle of Os atoms, two of which are bonded to four carbonyl ligands. The third Os atom is bound to three carbonyl ligands, and the arsine ligand [As(C_6_H_4_SCH_3_)_3_] occupies the equatorial position. In the crystal, the mol­ecules are linked by C—H⋯O hydrogen bonds. To further analyse the inter­molecular inter­actions, a Hirshfeld surface analysis was performed. Two out of the three methyl­sulfanyl groups are disordered over two sites with final refinement occupancy ratios of 0.612 (12):0.388 (12) and 0.620 (9):0.380 (9).

## Abstract 2

The title compound comprises a triangle of Os atoms, two of which are bonded to four carbonyl ligands. The third Os atom is bound to three carbonyl ligands, and the arsine ligand [As(C_6_H_4_SCH_3_)_3_] occupies the equatorial position. In the crystal, the mol­ecules are linked by C—H⋯O hydrogen bonds.

## 1. Chemical context

The chemistry of triosmium carbonyl clusters with group 15 ligands has been extensively studied (Raithby, 2024 ▸). The majority of reported structures have focused on tertiary phosphine ligands (P*R*_3_) with *R* = alkyl or aryl groups, with fewer examples involving tertiary arsine ligands (AsP*R*_3_) (webCSD, accessed May 2025; Groom *et al.*, 2016 ▸). These ligands stabilize low oxidation states of metal centres and can be used to modify both the electronic and steric properties of resulting coordination compounds (Honaker *et al.*, 2007 ▸). Numerous triosmium carbonyl clusters containing one, two, or three P*R*_3_ have been synthesized and fully characterised (Bruce *et al.*, 1988*a* ▸,*b* ▸; Biradha *et al.*, 2000 ▸). Os_3_(CO)_11_(P*R*_3_) is the most reported crystal structure among derivatives in this series, the earlier ones being Os_3_(CO)_11_(PPh_3_), and Os_3_(CO)_11_{PPh(OMe)_2_}, which were prepared in refluxing toluene for more than 10 h (Bruce *et al.*, 1988*a* ▸). Biradha and co-workers reported a series of Os_3_(CO)_11_(P*R*_3_) with *R* = F, OPh, Et, *p*-C_6_H_4_Me, *o*-C_6_H_4_Me, *p*-C_6_H_4_(CF_3_) and C_6_H_11_ (Biradha *et al.*, 2000 ▸) by reacting Os_3_(CO)_11_(CH_3_CN) with P*R*_3_ in di­chloro­methane for 15 min (Hansen *et al.*, 1998 ▸). According to these structures, the steric and electronic effects of P*R*_3_ often result in variations of the Os—Os bond that is *cis* to P*R*_3_ and the Os—P bond length (Biradha *et al.*, 2000 ▸). However, there are not many reactions conducted on Os_3_(CO)_12_ with AsP*R*_3_. Os_3_(CO)_11_(AsPh_3_) is the only reported structure in this series (Oh *et al.*, 2015 ▸). Thus, we are currently exploring the reaction between Os_3_(CO)_12_ and the AsP*R*_3_ ligand, focusing on how such ligands influence structural parameters within the triangular osmium cluster. We anti­cipate that the resulting compound will serve as a representative example contributing to the broader understanding of this structural series Os_3_(CO)_11_(P/As*R*_3_). Herein, we report the synthesis of Os_3_(CO)_11_{As(C_6_H_4_SCH_3_)_3_}, **1**, and its examination using single-crystal X-ray diffraction and Hirshfeld surface analysis.
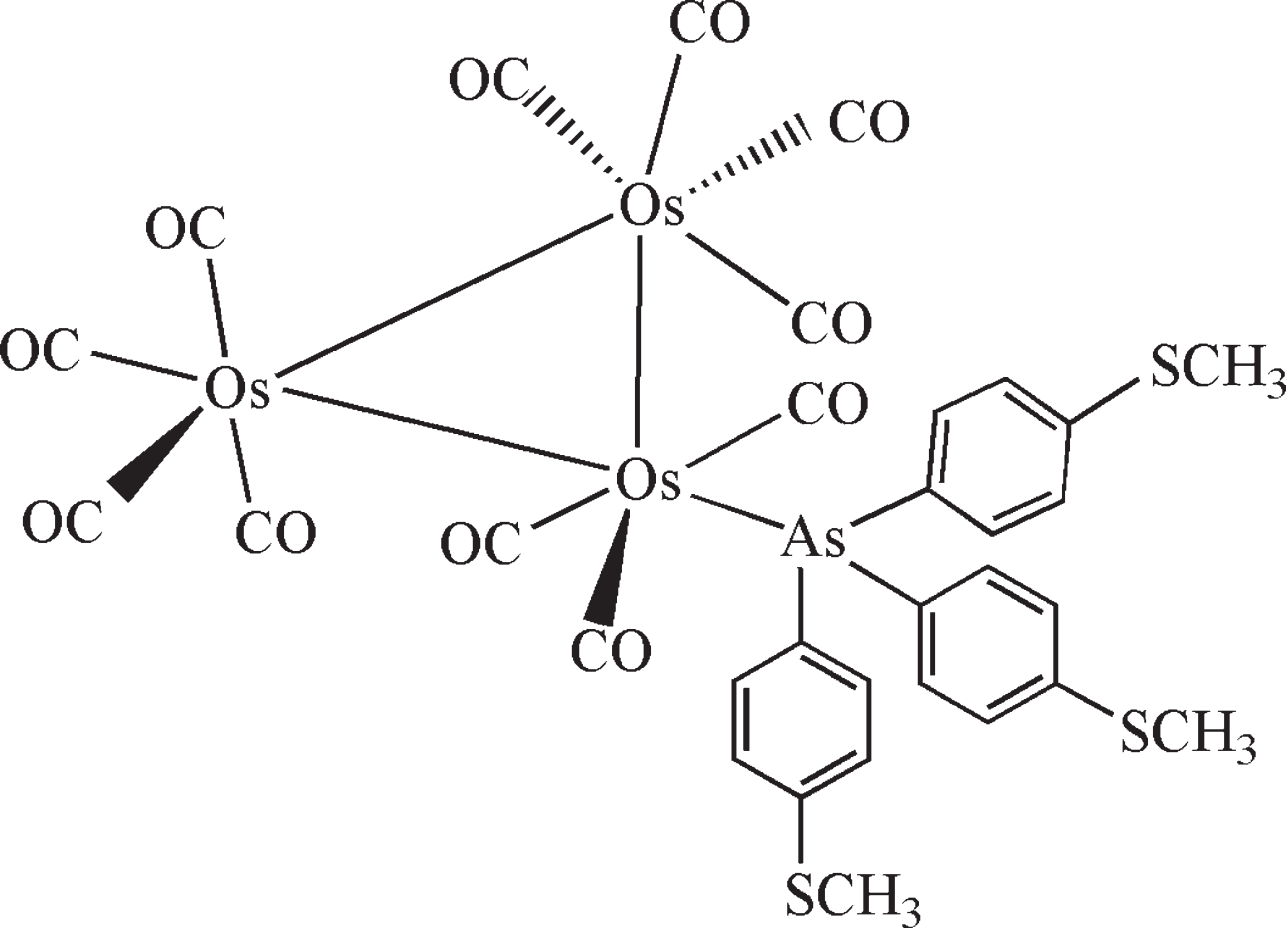


## 2. Structural commentary

**Figure 1:**
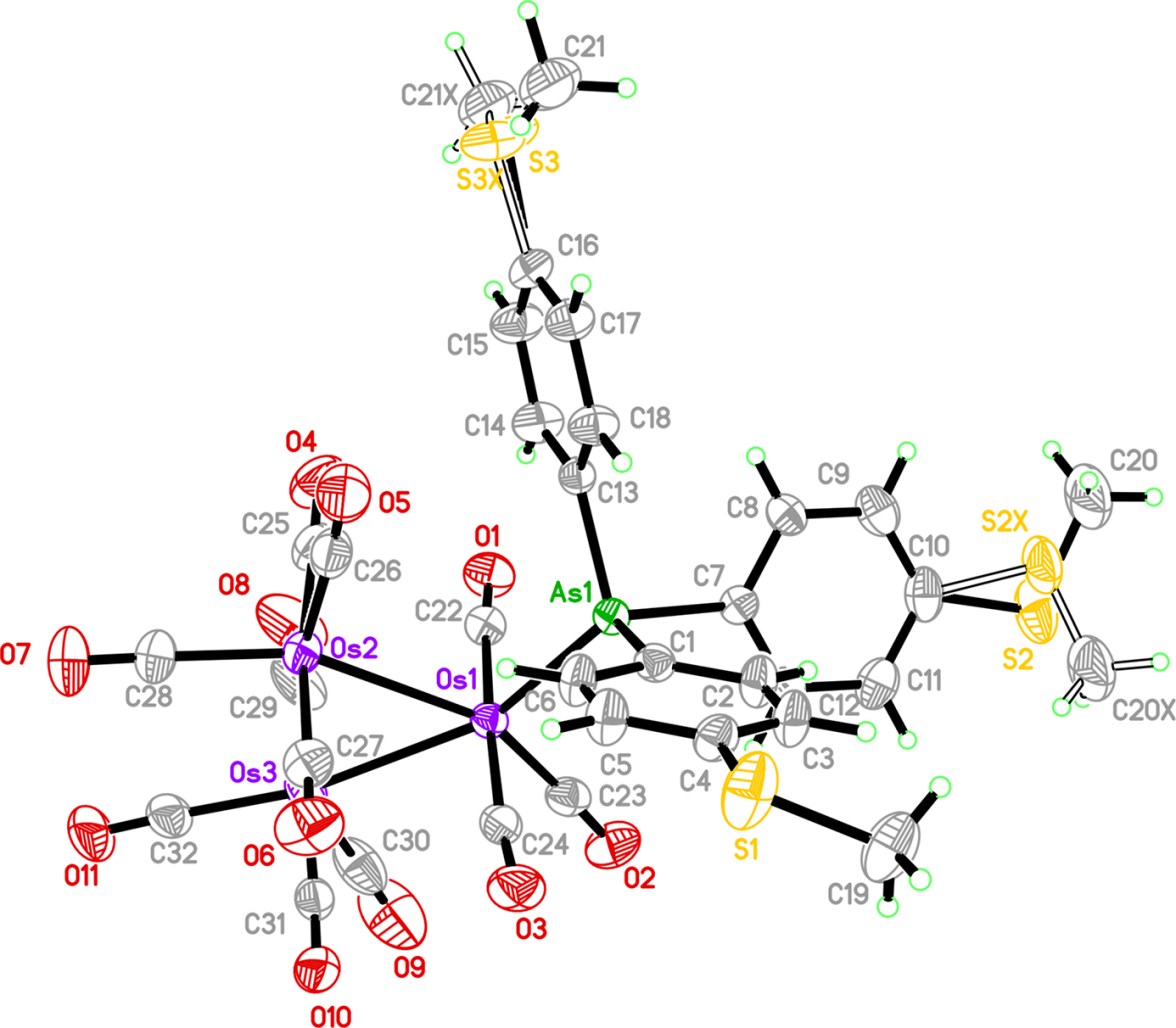
The mol­ecular structure of **1** with 30% displacement ellipsoids.

The mol­ecular structure of **1** is shown in Fig. 1 ▸. The mol­ecule comprises an Os_3_ triangle with one Os centre being equator­ially coordinated by the tris­{4-(methyl­sulfan­yl)phen­yl}arsine ligand [As(C_6_H_4_SCH_3_)_3_]. The Os—Os bond lengths in the Os_3_ triangle are not equivalent, with the Os1—Os2 bond [2.9150 (3) Å] being longer than the Os1—Os3 and Os2—Os3 bonds, at 2.8611 (3) and 2.8817 (4) Å, respectively. The longest Os—Os bond is *cis* to As(C_6_H_4_SCH_3_)_3_ and comparable with a similar Os—Os bond [2.9148 (7) Å] in Os_3_(CO)_11_AsPh_3_ (Oh *et al.*, 2015 ▸). These observations are attributed to the steric and electronic effects of the arsine ligand on the Os—Os bonds. The Os—As bond length in **1** is 2.4603 (5) Å and almost similar to the related bond in Os_3_(CO)_11_(AsPh_3_) 2.4670 (9) Å (Oh *et al.*, 2015 ▸). The Os—C(CO) bond lengths are in the range 1.872 (6)–1.951 (7) Å. The equatorial Os—C≡O bond angles range from 175.7 (5) to 178.4 (5)°, whereas the axial Os—C≡O bond angles range from 174.3 (6) to 177.2 (5)°.

## 3. Supra­molecular features

**Figure 2:**
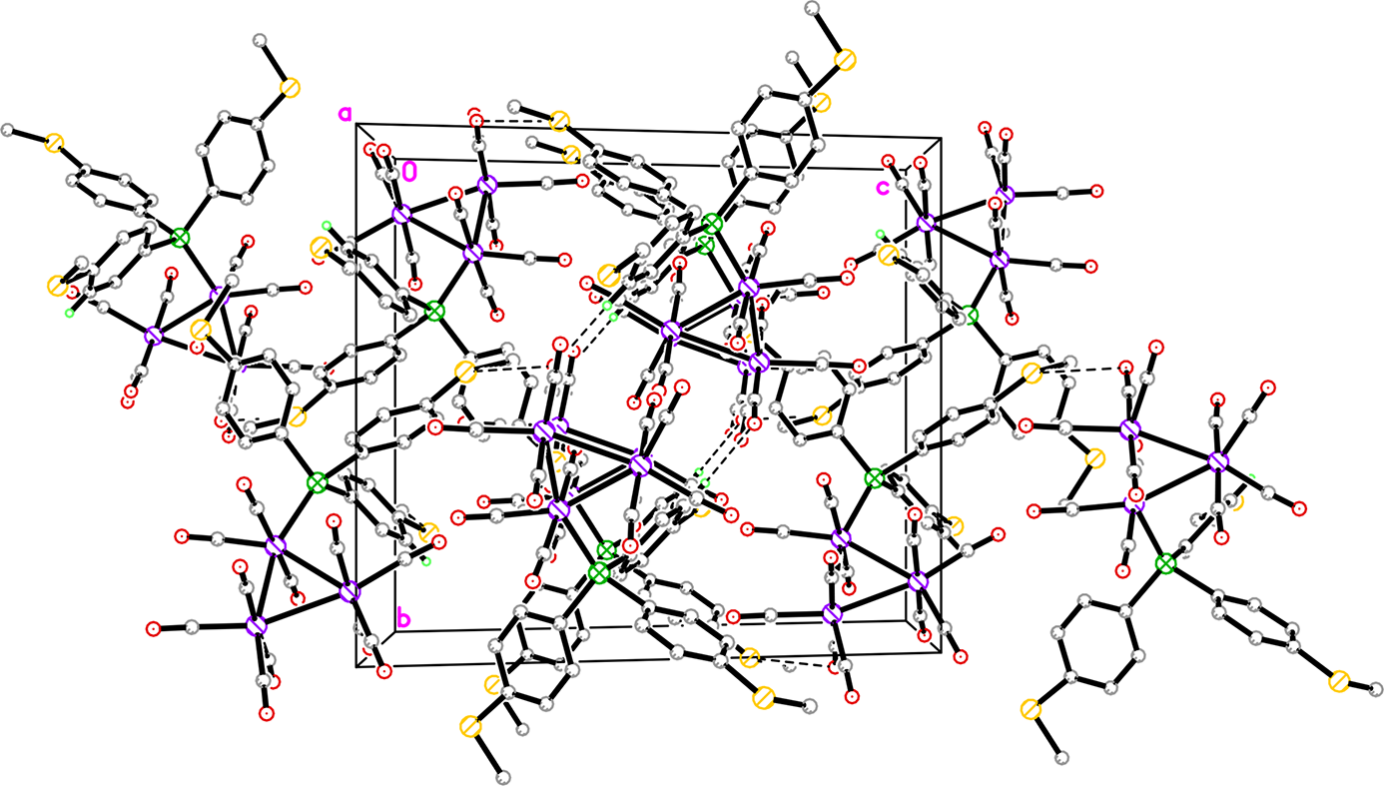
The crystal structure of the title compound viewed along the *c* axis. H atoms not involved in hydrogen bonds (dashed lines) have been omitted for clarity.

In the crystal, C5—H5*A*⋯O10 inter­molecular inter­actions (Table 1 ▸, Fig. 2 ▸) lead to the formation of an inversion dimer between two mol­ecules. This dimer is further consolidated by a short S3⋯O10 contact [3.21 (2) Å, symmetry code: 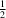 + *x*, 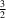 − *y*, 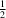 + *z*].

## 4. Hirshfeld surface analysis

The major and minor components of the disorder in **1** were subjected to Hirshfeld surface analysis using *CrystalExplorer21* (Spackman *et al.*, 2021 ▸). This is based on the procedures described in the literature (Tan *et al.*, 2019 ▸) to better understand the nature of the inter­molecular inter­actions that exist in the crystal.

**Figure 3:**
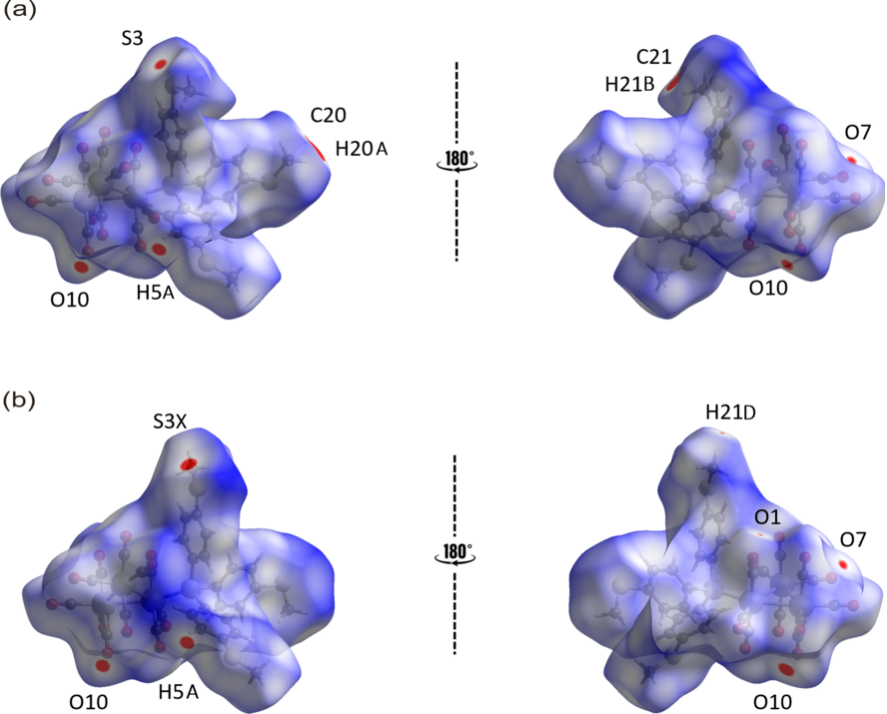
Two views of the *d*_norm_ map showing the atoms with close contacts, as indicated by the corresponding red spots, with varying intensities for (*a*) the major component and (*b*) the minor component of **1**.

The *d*_norm_ mapping reveals a relatively simple landscape of mol­ecular inter­actions, with only a few red spots being observed on the Hirshfeld surfaces. For the major component, the red spots originated from H5*A*, O10, H21*B*, C20, S3, O7, H20*A*, and C21 (Fig. 3 ▸*a*), corresponding to the close contacts for C5—H5*A*⋯O10, C21—H21*B*⋯C20, S3⋯O10, O7⋯O7, and C20—H20*A*⋯C21 (Table 2 ▸). The respective deviations of these contacts from the sum of the van der Waals radii are 0.20, 0.15, 0.11, 0.09 and 0.04 Å, reflecting the intensity of the corresponding red spots. For the minor component, red spots are detected on the disorder fragments of S3*X* and H21*D*, corresponding to S3*X*⋯O10 and C21*X*—H21*D*⋯O1 in addition to the intrinsic C5—H5*A*⋯O10 and O7⋯O7 close contacts for **1** (Fig. 3 ▸*b)*. The deviations between the *d*_norm_ contact distance and the sum of the van der Waals radii for S3*X*⋯O10 and H21*D*⋯O1 are 0.19 and 0.06 Å, respectively (Table 3 ▸).

**Figure 4:**
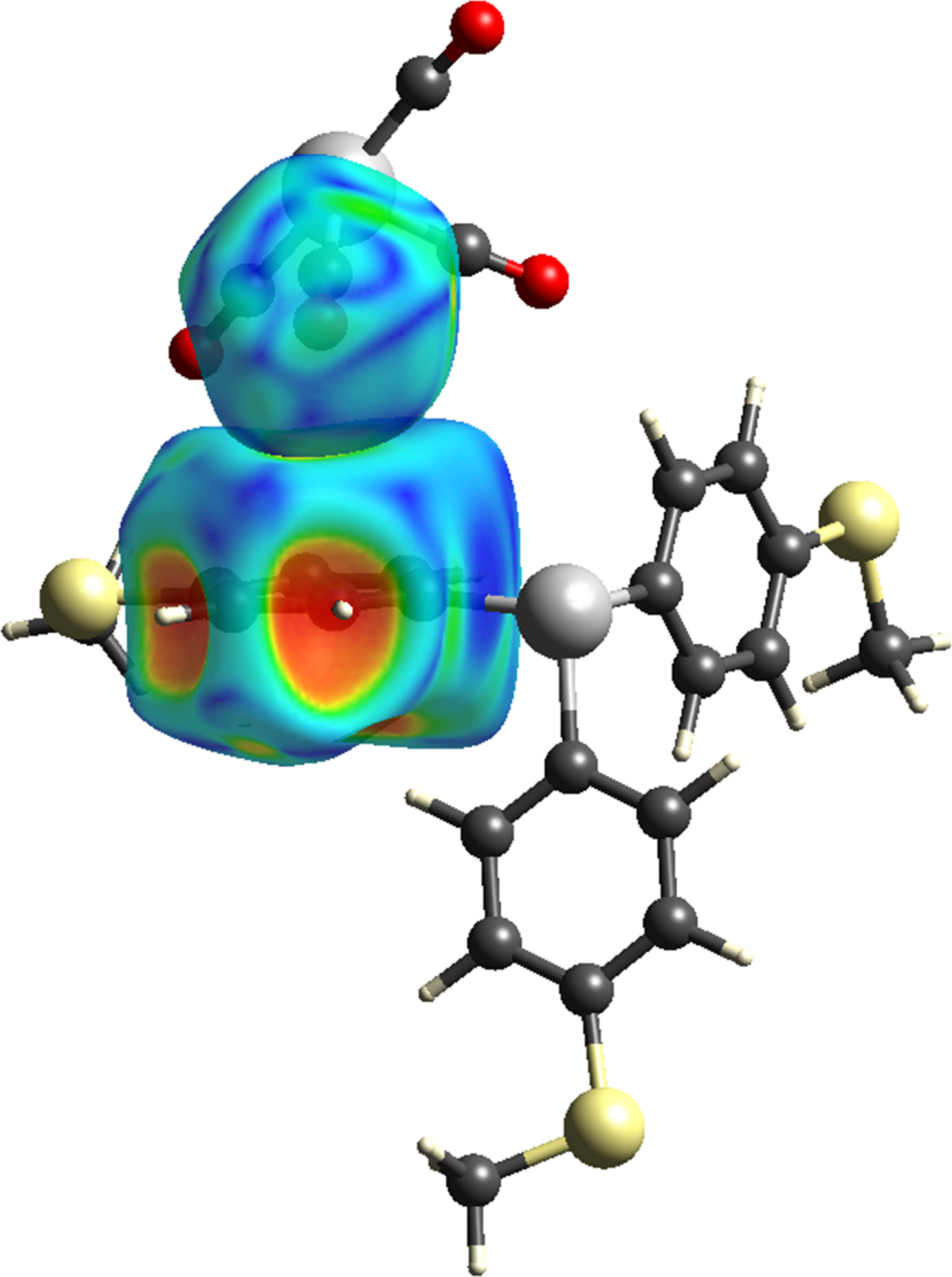
The partial Hirshfeld surface mapped with shape-index for C32–O11 (top) and the C7–C12 ring (bottom; 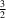 − *x*, 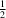 + *y*, 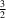 − *z*), showing the shape complementarity between the inter­molecular stacking fragments of **1**. The remaining parts of the mol­ecules were omitted for clarity.

Apart from the close contacts indicated by red spots, another significant feature emerges from the shape-index mapping on the Hirshfeld surface. This mapping highlights a complementary inter­molecular stacking inter­action between C32—O11 and the C7–C12 ring (symmetry code: 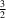 − *x*, 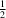 + *y*, 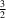 − *z*), providing evidence of a lone pair⋯π inter­action (Fig. 4 ▸). This finding aligns with the analysis from *PLATON* (Spek, 2020 ▸), which identified a contact distance of 3.867 (5) Å between O11 and the aromatic π-ring, along with a C≡O⋯π angle of 94.0 (4)°. Studies suggested that such inter­actions typically occur within a distance range of 3.0–4.5 Å, with angles varying between 60 and 160° (Caracelli *et al.*, 2016 ▸; Chen *et al.*, 2024 ▸), depending on the specific lone-pair donor and π-acceptor involved.

**Figure 5:**
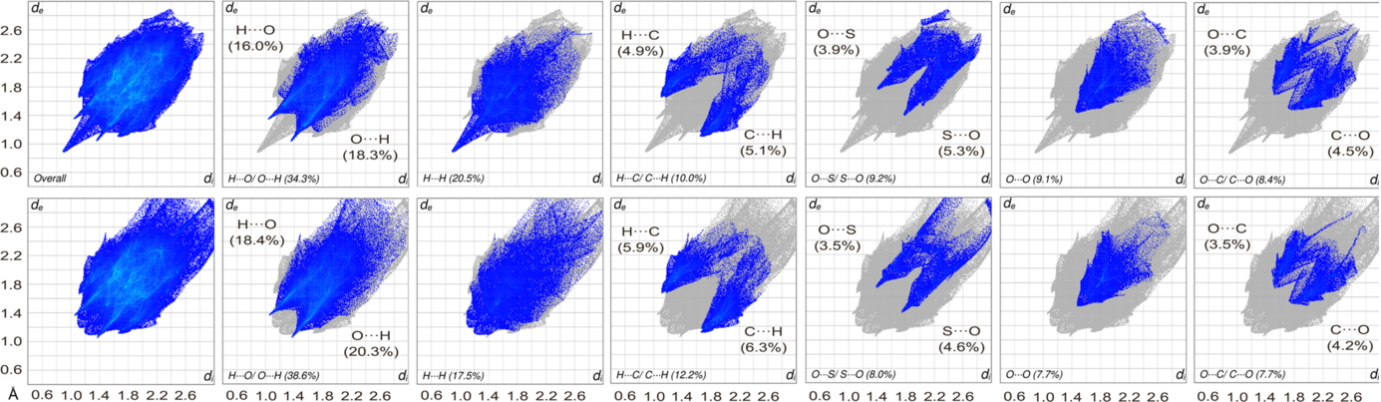
The comparison of the overall and prominent decomposed fingerprint print plots (> 5%) delineated into H⋯O/O⋯H, H⋯H, H⋯C/C⋯H, O⋯S/S⋯O, O⋯O, and O⋯C/C⋯O close contacts for the major (top) and minor (bottom) components of **1**.

The major and minor components of **1** were subjected to 2D fingerprint plot analysis to qu­anti­tatively assess close contacts within the structure. Both exhibit distinct shield- and paw-like fingerprint profiles, which, upon decomposition, reveal symmetrical H⋯O/O⋯H, H⋯H, H⋯C/C⋯H, O⋯S/S⋯O, O⋯O, and O⋯C/C⋯O inter­actions (Fig. 5 ▸), indicating homogeneous reciprocal contacts between inter­nal-*X*⋯*Y*-external and external-*X*⋯*Y*-inter­nal inter­faces.

**Figure 6:**
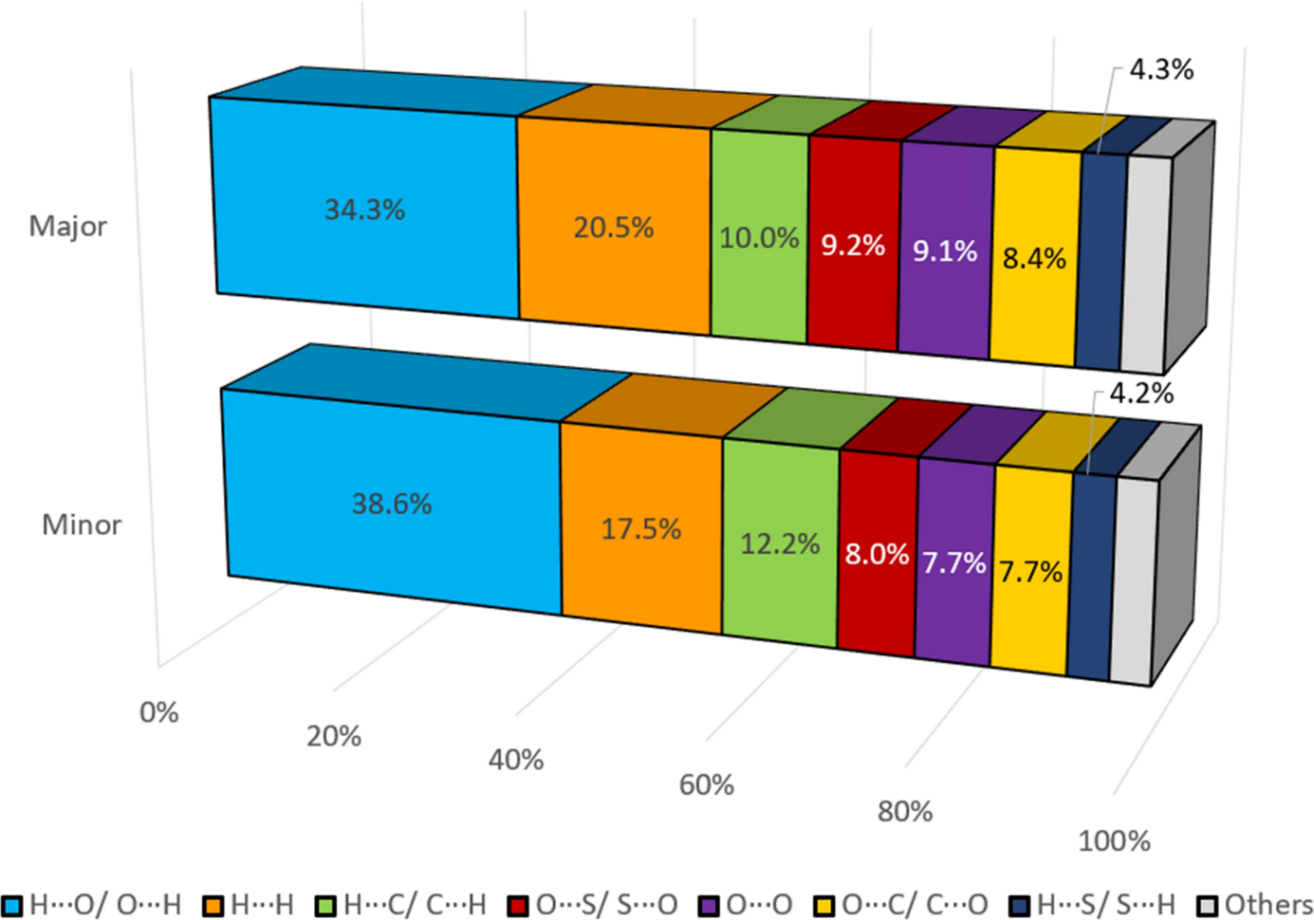
The percentage contributions of different close contacts to the Hirshfeld surfaces of the major and minor components of **1**.

Slight deviations are observed in the decomposed fingerprint plots of H⋯H and O⋯S/S⋯O. The major component features a distinct H⋯H peak at a *d*_i_ + *d*_e_ of approximately 1.80 Å (H20*A*⋯H21*A*), whereas the minor component exhibits a broader profile with a larger *d*_i_ + *d*_e_ of about 2.30 Å (H21*F*⋯H19*B*). In the pincer-like decomposed fingerprint plots of O⋯S/S⋯O, the reciprocal contacts correspond to O10⋯S3 (*d*_i_ + *d*_e_ ≃ 3.21 Å) in the major component and O10⋯S3*X* (*d*_i_ + *d*_e_ ≃ 3.12 Å) in the minor component. Other fingerprint plots remain consistent across both components, with close contact distributions being as follows: H⋯O/O⋯H (34.3% *vs* 38.6%), H⋯H (20.5% *vs* 17.5%), H⋯C/C⋯H (10.0% *vs* 12.2%), O⋯S/S⋯O (9.2% *vs* 8.0%), O⋯O (9.1% *vs* 7.7%), and O⋯C/C⋯O (8.4% *vs* 7.7%), while the remaining less prominent contacts contribute to approximately 4.1% to 4.2% (Fig. 6 ▸).

## 5. Database survey

A search of the Cambridge Structural Database (webCSD accessed May 2025; Groom *et al.*, 2016 ▸) for the title compound returned no relevant hits. However, a search with generalized tertiary arsine ligand (As*R*_3_) returned one hit [Os_3_(CO)_11_(AsPh_3_)] [CSD refcode YUCXEB; Oh *et al.*, 2015 ▸). The structure of the title compound is very similar to that of Ru_3_(CO)_11_{As(C_6_H_4_SCH_3_)_3_} (SUXQEI; Shawkataly *et al.*, 2010 ▸), in which tris­{4-(methyl­sulfan­yl)phen­yl}arsine [As(C_6_H_4_SCH_3_)_3_] is equatorially bonded to the metal centre. The structure is consistent with literature precedents for complexes with the general formula Os_3_(CO)_11_P*R*_3_ [HIYVUG (Hansen *et al.*, 1998 ▸), MASPEB, MASPIF and MASPUR (Biradha *et al.*, 2000 ▸), VADYEE (Bruce *et al.*, 1988*a* ▸, VAWWUL (Ang *et al.*, 1989 ▸), YEDZOW and YEFCAN (Adams *et al.*, 1994 ▸)].

## 6. Synthesis and crystallization

A solution of Os_3_(CO)_11_(CH_3_CN) (Nicholls *et al.*, 1990 ▸; Hansen *et al.*, 1998 ▸) (80 mg, 0.087 mmol) and As(C_6_H_4_SCH_3_)_3_ (60 mg, 0.13 mmol) in CH_2_Cl_2_ (25 ml) was stirred at room temperature for 60 min. The reaction was carried out under nitro­gen-free oxygen using standard Schlenk techniques. The solution was dried in *vacuo*, and the residue was chromatographed by preparative thin-layer chromatography on silica gel with C_6_H_14_/CH_2_Cl_2_ (3:2) as the eluent. Chromatographic separation afforded two bands: the first yellow band was identified as the starting material Os_3_(CO)_12_. The desired product Os_3_(CO)_11_{As(C_6_H_4_SCH_3_)_3_} was isolated as the second yellow band in 50% yield and recrystallized from CH_2_Cl_2_/CH_3_OH (1:3). IR (ATR): ν(CO) 2107 *s*, 2051 *m*, 2006 *w*, 1974 *m*, 1942 *w* cm^−1^.

## 7. Refinement

Crystal data, data collection and structure refinement details are summarized in Table 3 ▸. All H atoms were positioned geometrically and refined using a riding model with C—H distances of 0.93 or 0.96 Å (for methyl groups) and *U*_iso_(H) = 1.2*U*_eq_(C) or 1.5U_eq_(C-meth­yl). Two out of the three methyl­sulfanyl groups were disordered over two sites with a final refinement occupancy ratios of 0.612 (12):0.388 (12) and 0.620 (9):0.380 (9). Anisotropic displacement parameters and rigid-body restraints were applied to the disordered components. Due to poor agreement, reflections 020, 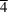
33, 680, 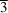
07, and 379 were omitted from the final cycles of refinement. Maximum and minimum residual electron densities of 1.46 and −1.46 e Å^−3^ were observed at 0.83 Å from Os1 and 0.70 Å from Os3, respectively.

## Supplementary Material

Crystal structure: contains datablock(s) I. DOI: 10.1107/S2056989025006541/tx2100sup1.cif

Structure factors: contains datablock(s) I. DOI: 10.1107/S2056989025006541/tx2100Isup2.hkl

CCDC reference: 2474325

Additional supporting information:  crystallographic information; 3D view; checkCIF report

## Figures and Tables

**Table 1:** Hydrogen-bond geometry (Å, °)

*D*—H⋯*A*	*D*—H	H⋯*A*	*D*⋯*A*	*D*—H⋯*A*
C5—H5*A*⋯O10^i^	0.93	2.55	3.41 (1)	154

Symmetry code: (i) 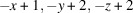.

**Table 2:** The *d*_norm_ contact distances (Å, adjusted to neutron values) for all identified inter­actions present in the major and minor components of **1** with respect to the corresponding sum of van der Waals radii of the relevant contact atoms

Contact	*d*_norm_ Distance	ΣvdW radii	Δ(ΣvdW – *d*_norm_ distance)	Symmetry operation
**Major**				
C5–H5*A*⋯O10	2.41	2.61	0.20	1 − *x*, 2 − *y*, 2 − *z*
C20–H20*A*⋯C21	2.57	2.61	0.04	− 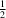 + *x*, 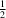 − *y*, − 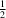 + *z*
C21–H21*B*⋯C20	2.64	2.79	0.15	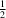 + *x*, 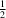 − *y*, 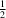 + *z*
S3⋯O10	3.21	3.32	0.11	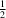 + *x*, 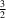 − *y*, 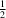 + *z*
O7⋯O7	2.95	3.04	0.09	2 − *x*, 2 − *y*, 2 − *z*
**Minor**				
C5–H5*A*⋯O10	2.41	2.61	0.20	1 − *x*, 2 − *y*, 2 − *z*
S3*X*⋯O10	3.13	3.32	0.19	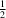 + *x*, 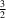 − *y*, 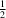 + *z*
O7⋯O7	2.95	3.04	0.09	2 − *x*, 2 − *y*, 2 − *z*
C21*X*–H21*D*⋯O1	2.55	2.61	0.06	2 − *x*, 1 − *y*, 2 − *z*

**Table 3:** Experimental details

Crystal data	
Chemical formula	[Os_3_(C_21_H_21_AsS_3_)(CO)_11_]
*M*_r_	1323.19
Crystal system, space group	Monoclinic, *P*2_1_/*n*
Temperature (K)	297
*a*, *b*, *c* (Å)	14.5019 (15), 15.3759 (16), 17.8575 (19)
β (°)	107.870 (2)
*V* (Å^3^)	3789.8 (7)
*Z*	4
Radiation type	Mo *K*α
μ (mm^−1^)	11.12
Crystal size (mm)	0.41 × 0.15 × 0.09

Data collection	
Diffractometer	Bruker *APEX* Duo CCD area detector
Absorption correction	Multi-scan (*SADABS*; Krause et al., 2015 ▸)
*T*_min_, *T*_max_	0.024, 0.076
No. of measured, independent and observed [*I* > 2σ(*I*)] reflections	52142, 13246, 8766
*R*_int_	0.054
(sin θ/λ)_max_ (Å^−1^)	0.749

Refinement	
*R*[*F*^2^ > 2σ(*F*^2^)], *wR*(*F*^2^), *S*	0.033, 0.081, 0.97
No. of reflections	13246
No. of parameters	482
No. of restraints	64
H-atom treatment	H-atom parameters constrained
Δρ_max_, Δρ_min_ (e Å^−3^)	1.46, −1.46

Computer programs: *APEX2* and *SAINT* (Bruker, 2012 ▸), *SHELXT* (Sheldrick, 2015*a* ▸), *SHELXL2014/7* (Sheldrick, 2015*b* ▸), *SHELXTL* (Sheldrick, 2015*a* ▸) and *PLATON* (Spek, 2020 ▸).

## supplementary crystallographic information

### Undecacarbonyl{tris[4-(methylsulfanyl)phenyl]arsine}-triangulo-triosmium(0) Crystal data

**Table:**

[Os_3_(C_21_H_21_AsS_3_)(CO)_11_]	*F*(000) = 2440
*M**_r_* = 1323.19	*D*_x_ = 2.319 Mg m^−^^3^
Monoclinic, *P*2_1_/*n*	Mo *K*α radiation, λ = 0.71073 Å
*a* = 14.5019 (15) Å	Cell parameters from 9975 reflections
*b* = 15.3759 (16) Å	θ = 2.7–27.7°
*c* = 17.8575 (19) Å	µ = 11.12 mm^−^^1^
β = 107.870 (2)°	*T* = 297 K
*V* = 3789.8 (7) Å^3^	Plate, yellow
*Z* = 4	0.41 × 0.15 × 0.09 mm

### Undecacarbonyl{tris[4-(methylsulfanyl)phenyl]arsine}-triangulo-triosmium(0) Data collection

**Table:**

Bruker APEX Duo CCD area detector diffractometer	8766 reflections with *I* > 2σ(*I*)
Radiation source: fine-focus sealed tube	*R*_int_ = 0.054
φ and ω scans	θ_max_ = 32.2°, θ_min_ = 2.7°
Absorption correction: multi-scan (SADABS; Krause et al., 2015)	*h* = −20→21
*T*_min_ = 0.024, *T*_max_ = 0.076	*k* = −22→23
52142 measured reflections	*l* = −25→26
13246 independent reflections	

### Undecacarbonyl{tris[4-(methylsulfanyl)phenyl]arsine}-triangulo-triosmium(0) Refinement

**Table:**

Refinement on *F*^2^	64 restraints
Least-squares matrix: full	Hydrogen site location: inferred from neighbouring sites
*R*[*F*^2^ > 2σ(*F*^2^)] = 0.033	H-atom parameters constrained
*wR*(*F*^2^) = 0.081	*w* = 1/[σ^2^(*F*_o_^2^) + (0.0331*P*)^2^] where *P* = (*F*_o_^2^ + 2*F*_c_^2^)/3
*S* = 0.97	(Δ/σ)_max_ = 0.003
13246 reflections	Δρ_max_ = 1.46 e Å^−^^3^
482 parameters	Δρ_min_ = −1.46 e Å^−^^3^

### Undecacarbonyl{tris[4-(methylsulfanyl)phenyl]arsine}-triangulo-triosmium(0) Special details

**Table:**

Geometry. All esds (except the esd in the dihedral angle between two l.s. planes) are estimated using the full covariance matrix. The cell esds are taken into account individually in the estimation of esds in distances, angles and torsion angles; correlations between esds in cell parameters are only used when they are defined by crystal symmetry. An approximate (isotropic) treatment of cell esds is used for estimating esds involving l.s. planes.

### Undecacarbonyl{tris[4-(methylsulfanyl)phenyl]arsine}-triangulo-triosmium(0) Fractional atomic coordinates and isotropic or equivalent isotropic displacement parameters (Å^2^)

**Table:**

	*x*	*y*	*z*	*U*_iso_*/*U*_eq_	Occ. (<1)
Os1	0.64030 (2)	0.79000 (2)	0.83741 (2)	0.04272 (5)	
Os2	0.77751 (2)	0.87250 (2)	0.97335 (2)	0.05053 (6)	
Os3	0.75404 (2)	0.93459 (2)	0.81633 (2)	0.05645 (6)	
As1	0.58467 (3)	0.66988 (3)	0.90375 (2)	0.04018 (9)	
S1	0.26975 (11)	0.78176 (13)	1.07849 (11)	0.0909 (6)	
S2	0.3328 (6)	0.3694 (4)	0.6797 (4)	0.098 (2)	0.612 (12)
C20	0.3547 (12)	0.2721 (10)	0.7360 (9)	0.126 (4)	0.612 (12)
H20A	0.3099	0.2281	0.7089	0.189*	0.612 (12)
H20B	0.3465	0.2829	0.7865	0.189*	0.612 (12)
H20C	0.4197	0.2528	0.7429	0.189*	0.612 (12)
S2X	0.3528 (7)	0.3392 (6)	0.7063 (7)	0.089 (3)	0.388 (12)
C20X	0.252 (2)	0.3784 (14)	0.6533 (15)	0.126 (4)	0.388 (12)
H20D	0.2008	0.3392	0.6530	0.189*	0.388 (12)
H20E	0.2544	0.3862	0.6005	0.189*	0.388 (12)
H20F	0.2414	0.4336	0.6743	0.189*	0.388 (12)
S3	0.9056 (13)	0.4559 (13)	1.1746 (11)	0.102 (4)	0.620 (9)
C21	0.8517 (8)	0.4338 (9)	1.2521 (7)	0.104 (3)	0.620 (9)
H21A	0.8994	0.4082	1.2963	0.156*	0.620 (9)
H21B	0.7984	0.3943	1.2329	0.156*	0.620 (9)
H21C	0.8289	0.4872	1.2680	0.156*	0.620 (9)
S3X	0.905 (2)	0.4698 (19)	1.1841 (16)	0.083 (4)	0.380 (9)
C21X	0.9804 (14)	0.4194 (15)	1.1371 (12)	0.104 (3)	0.380 (9)
H21D	1.0389	0.4004	1.1757	0.156*	0.380 (9)
H21E	0.9960	0.4598	1.1019	0.156*	0.380 (9)
H21F	0.9474	0.3701	1.1079	0.156*	0.380 (9)
O1	0.7863 (3)	0.6672 (3)	0.7993 (2)	0.0725 (10)	
O2	0.5161 (3)	0.7732 (3)	0.6676 (2)	0.0875 (13)	
O3	0.4744 (3)	0.9005 (3)	0.8608 (3)	0.0838 (12)	
O4	0.9236 (3)	0.7338 (4)	0.9578 (3)	0.1041 (16)	
O5	0.7750 (4)	0.7808 (3)	1.1233 (3)	0.0938 (14)	
O6	0.6178 (4)	0.9914 (3)	0.9960 (3)	0.0998 (15)	
O7	0.9269 (3)	1.0144 (3)	1.0409 (3)	0.0917 (14)	
O8	0.9216 (5)	0.8158 (4)	0.8063 (4)	0.136 (2)	
O9	0.6690 (7)	0.9423 (4)	0.6384 (3)	0.152 (3)	
O10	0.5906 (3)	1.0542 (3)	0.8289 (2)	0.0752 (11)	
O11	0.8903 (3)	1.0898 (3)	0.8460 (3)	0.0991 (15)	
C1	0.4875 (3)	0.6994 (3)	0.9544 (3)	0.0444 (9)	
C2	0.4001 (3)	0.6577 (3)	0.9364 (3)	0.0555 (12)	
H2A	0.3860	0.6135	0.8992	0.067*	
C3	0.3318 (3)	0.6811 (4)	0.9734 (3)	0.0598 (12)	
H3A	0.2727	0.6520	0.9605	0.072*	
C4	0.3507 (3)	0.7467 (3)	1.0290 (3)	0.0520 (11)	
C5	0.4391 (3)	0.7880 (3)	1.0464 (3)	0.0583 (12)	
H5A	0.4534	0.8322	1.0838	0.070*	
C6	0.5069 (3)	0.7657 (3)	1.0100 (3)	0.0538 (11)	
H6A	0.5658	0.7951	1.0226	0.065*	
C7	0.5209 (3)	0.5744 (3)	0.8363 (2)	0.0442 (9)	
C8	0.5417 (4)	0.4876 (3)	0.8579 (3)	0.0527 (10)	
H8A	0.5917	0.4744	0.9034	0.063*	
C9	0.4893 (4)	0.4213 (4)	0.8128 (3)	0.0666 (14)	
H9A	0.5053	0.3638	0.8272	0.080*	
C10	0.4135 (4)	0.4392 (4)	0.7466 (3)	0.0651 (14)	
C11	0.3932 (4)	0.5262 (4)	0.7243 (3)	0.0690 (14)	
H11A	0.3424	0.5395	0.6794	0.083*	
C12	0.4478 (4)	0.5918 (4)	0.7684 (3)	0.0582 (12)	
H12A	0.4350	0.6492	0.7520	0.070*	
C13	0.6804 (3)	0.6093 (3)	0.9873 (2)	0.0421 (9)	
C14	0.7659 (4)	0.5830 (4)	0.9766 (3)	0.0617 (13)	
H14A	0.7786	0.5965	0.9299	0.074*	
C15	0.8332 (4)	0.5369 (4)	1.0342 (3)	0.0682 (14)	
H15A	0.8899	0.5181	1.0252	0.082*	
C16	0.8178 (3)	0.5182 (4)	1.1049 (3)	0.0589 (12)	
C17	0.7331 (4)	0.5453 (3)	1.1170 (3)	0.0615 (13)	
H17A	0.7214	0.5336	1.1644	0.074*	
C18	0.6649 (4)	0.5903 (3)	1.0575 (3)	0.0559 (11)	
H18A	0.6074	0.6080	1.0657	0.067*	
C19	0.1568 (4)	0.7396 (5)	1.0225 (4)	0.090 (2)	
H19A	0.1085	0.7550	1.0468	0.135*	
H19B	0.1610	0.6774	1.0198	0.135*	
H19C	0.1394	0.7634	0.9703	0.135*	
C22	0.7360 (4)	0.7139 (3)	0.8156 (3)	0.0518 (10)	
C23	0.5636 (4)	0.7775 (3)	0.7325 (3)	0.0603 (12)	
C24	0.5387 (4)	0.8629 (3)	0.8546 (3)	0.0536 (11)	
C25	0.8686 (4)	0.7860 (4)	0.9595 (3)	0.0687 (15)	
C26	0.7747 (4)	0.8120 (4)	1.0660 (4)	0.0670 (14)	
C27	0.6752 (4)	0.9480 (4)	0.9840 (3)	0.0648 (13)	
C28	0.8726 (4)	0.9610 (4)	1.0160 (3)	0.0653 (13)	
C29	0.8587 (6)	0.8578 (4)	0.8105 (5)	0.094 (2)	
C30	0.7036 (7)	0.9381 (4)	0.7049 (4)	0.095 (2)	
C31	0.6500 (4)	1.0078 (3)	0.8269 (3)	0.0557 (11)	
C32	0.8418 (4)	1.0322 (4)	0.8361 (4)	0.0701 (15)	

### Undecacarbonyl{tris[4-(methylsulfanyl)phenyl]arsine}-triangulo-triosmium(0) Atomic displacement parameters (Å^2^)

**Table:**

	*U*^11^	*U*^22^	*U*^33^	*U*^12^	*U*^13^	*U*^23^
Os1	0.04402 (9)	0.04182 (9)	0.04327 (10)	0.00121 (7)	0.01478 (7)	0.00311 (6)
Os2	0.04408 (9)	0.05623 (11)	0.05050 (11)	−0.00492 (8)	0.01333 (7)	−0.00148 (8)
Os3	0.06879 (12)	0.04738 (10)	0.06249 (13)	−0.00426 (9)	0.03387 (10)	0.00544 (8)
As1	0.0409 (2)	0.0400 (2)	0.0403 (2)	0.00060 (16)	0.01330 (17)	−0.00004 (16)
S1	0.0575 (8)	0.1229 (15)	0.1047 (13)	−0.0127 (8)	0.0434 (8)	−0.0478 (11)
S2	0.127 (5)	0.084 (4)	0.081 (3)	−0.049 (4)	0.029 (3)	−0.031 (2)
C20	0.144 (9)	0.087 (7)	0.124 (9)	−0.049 (7)	0.006 (8)	−0.023 (6)
S2X	0.094 (4)	0.058 (5)	0.114 (7)	−0.028 (3)	0.028 (4)	−0.025 (3)
C20X	0.144 (9)	0.087 (7)	0.124 (9)	−0.049 (7)	0.006 (8)	−0.023 (6)
S3	0.070 (4)	0.142 (10)	0.075 (5)	0.025 (5)	−0.007 (3)	0.036 (5)
C21	0.074 (5)	0.134 (9)	0.090 (7)	0.001 (5)	0.005 (4)	0.032 (6)
S3X	0.079 (7)	0.097 (5)	0.066 (6)	0.032 (4)	0.014 (5)	0.027 (5)
C21X	0.074 (5)	0.134 (9)	0.090 (7)	0.001 (5)	0.005 (4)	0.032 (6)
O1	0.076 (2)	0.069 (2)	0.081 (3)	0.018 (2)	0.037 (2)	−0.0059 (19)
O2	0.089 (3)	0.111 (4)	0.047 (2)	0.008 (3)	−0.001 (2)	0.008 (2)
O3	0.067 (2)	0.075 (3)	0.117 (4)	0.022 (2)	0.040 (2)	0.009 (2)
O4	0.065 (2)	0.116 (4)	0.117 (4)	0.026 (3)	0.007 (3)	−0.020 (3)
O5	0.107 (3)	0.103 (3)	0.062 (3)	−0.028 (3)	0.013 (2)	0.019 (2)
O6	0.098 (3)	0.112 (4)	0.095 (3)	0.033 (3)	0.038 (3)	−0.018 (3)
O7	0.086 (3)	0.099 (3)	0.097 (3)	−0.044 (3)	0.038 (2)	−0.028 (3)
O8	0.134 (4)	0.092 (4)	0.239 (7)	0.029 (3)	0.141 (5)	0.042 (4)
O9	0.286 (9)	0.112 (4)	0.062 (4)	−0.028 (5)	0.059 (5)	−0.002 (3)
O10	0.065 (2)	0.065 (2)	0.083 (3)	0.003 (2)	0.005 (2)	−0.0114 (19)
O11	0.090 (3)	0.070 (3)	0.143 (4)	−0.025 (2)	0.043 (3)	0.016 (3)
C1	0.045 (2)	0.041 (2)	0.049 (2)	0.0043 (17)	0.0162 (18)	−0.0003 (17)
C2	0.048 (2)	0.059 (3)	0.061 (3)	−0.012 (2)	0.019 (2)	−0.019 (2)
C3	0.044 (2)	0.068 (3)	0.072 (3)	−0.018 (2)	0.024 (2)	−0.017 (2)
C4	0.042 (2)	0.060 (3)	0.057 (3)	0.003 (2)	0.019 (2)	−0.004 (2)
C5	0.052 (2)	0.063 (3)	0.067 (3)	−0.010 (2)	0.027 (2)	−0.022 (2)
C6	0.043 (2)	0.062 (3)	0.060 (3)	−0.012 (2)	0.020 (2)	−0.015 (2)
C7	0.047 (2)	0.048 (2)	0.038 (2)	0.0006 (18)	0.0132 (17)	−0.0042 (16)
C8	0.062 (3)	0.044 (2)	0.054 (3)	−0.001 (2)	0.021 (2)	−0.0018 (19)
C9	0.084 (4)	0.053 (3)	0.070 (4)	−0.008 (3)	0.036 (3)	−0.013 (2)
C10	0.069 (3)	0.067 (3)	0.067 (4)	−0.020 (3)	0.032 (3)	−0.028 (3)
C11	0.065 (3)	0.087 (4)	0.050 (3)	−0.010 (3)	0.010 (2)	−0.018 (3)
C12	0.067 (3)	0.058 (3)	0.047 (3)	−0.001 (2)	0.012 (2)	−0.001 (2)
C13	0.043 (2)	0.041 (2)	0.042 (2)	−0.0020 (16)	0.0122 (17)	−0.0006 (16)
C14	0.057 (3)	0.077 (4)	0.057 (3)	0.014 (2)	0.027 (2)	0.014 (2)
C15	0.052 (3)	0.083 (4)	0.074 (4)	0.019 (3)	0.025 (3)	0.017 (3)
C16	0.046 (2)	0.068 (3)	0.052 (3)	0.002 (2)	−0.001 (2)	0.005 (2)
C17	0.069 (3)	0.069 (3)	0.042 (3)	0.001 (3)	0.010 (2)	0.008 (2)
C18	0.057 (3)	0.065 (3)	0.049 (3)	0.012 (2)	0.021 (2)	0.010 (2)
C19	0.049 (3)	0.130 (6)	0.100 (5)	−0.005 (3)	0.037 (3)	−0.012 (4)
C22	0.056 (2)	0.055 (3)	0.047 (3)	0.000 (2)	0.020 (2)	−0.0001 (19)
C23	0.064 (3)	0.058 (3)	0.061 (3)	0.006 (2)	0.022 (3)	0.012 (2)
C24	0.052 (2)	0.047 (3)	0.064 (3)	0.002 (2)	0.020 (2)	0.004 (2)
C25	0.044 (2)	0.080 (4)	0.076 (4)	0.001 (3)	0.009 (2)	−0.012 (3)
C26	0.057 (3)	0.073 (4)	0.065 (4)	−0.017 (3)	0.010 (3)	0.001 (3)
C27	0.071 (3)	0.073 (4)	0.051 (3)	0.000 (3)	0.019 (3)	−0.003 (2)
C28	0.058 (3)	0.075 (4)	0.065 (3)	−0.015 (3)	0.022 (3)	−0.011 (3)
C29	0.106 (5)	0.061 (4)	0.152 (7)	0.002 (3)	0.094 (5)	0.016 (4)
C30	0.162 (7)	0.064 (4)	0.072 (5)	−0.015 (4)	0.054 (5)	−0.001 (3)
C31	0.059 (3)	0.050 (3)	0.054 (3)	−0.007 (2)	0.012 (2)	−0.002 (2)
C32	0.068 (3)	0.063 (3)	0.084 (4)	−0.003 (3)	0.029 (3)	0.016 (3)

### Undecacarbonyl{tris[4-(methylsulfanyl)phenyl]arsine}-triangulo-triosmium(0) Geometric parameters (Å, º)

**Table:**

Os1—C23	1.872 (6)	O3—C24	1.131 (6)
Os1—C22	1.944 (5)	O4—C25	1.139 (7)
Os1—C24	1.950 (5)	O5—C26	1.129 (7)
Os1—As1	2.4603 (5)	O6—C27	1.137 (7)
Os1—Os3	2.8611 (3)	O7—C28	1.130 (6)
Os1—Os2	2.9150 (3)	O8—C29	1.139 (7)
Os2—C26	1.910 (6)	O9—C30	1.142 (8)
Os2—C28	1.921 (6)	O10—C31	1.128 (6)
Os2—C27	1.939 (6)	O11—C32	1.112 (7)
Os2—C25	1.943 (6)	C1—C2	1.368 (6)
Os2—Os3	2.8817 (4)	C1—C6	1.389 (6)
Os3—C30	1.898 (8)	C2—C3	1.396 (7)
Os3—C32	1.929 (6)	C2—H2A	0.9300
Os3—C31	1.938 (6)	C3—C4	1.383 (7)
Os3—C29	1.951 (7)	C3—H3A	0.9300
As1—C13	1.936 (4)	C4—C5	1.377 (6)
As1—C7	1.943 (4)	C5—C6	1.379 (6)
As1—C1	1.948 (4)	C5—H5A	0.9300
S1—C4	1.757 (5)	C6—H6A	0.9300
S1—C19	1.760 (6)	C7—C12	1.369 (6)
S2—C10	1.759 (8)	C7—C8	1.397 (6)
S2—C20	1.775 (17)	C8—C9	1.376 (7)
C20—H20A	0.9600	C8—H8A	0.9300
C20—H20B	0.9600	C9—C10	1.372 (8)
C20—H20C	0.9600	C9—H9A	0.9300
S2X—C20X	1.59 (3)	C10—C11	1.400 (8)
S2X—C10	1.809 (10)	C11—C12	1.373 (7)
C20X—H20D	0.9600	C11—H11A	0.9300
C20X—H20E	0.9600	C12—H12A	0.9300
C20X—H20F	0.9600	C13—C14	1.372 (6)
S3—C16	1.765 (16)	C13—C18	1.372 (6)
S3—C21	1.82 (2)	C14—C15	1.377 (7)
C21—H21A	0.9600	C14—H14A	0.9300
C21—H21B	0.9600	C15—C16	1.380 (7)
C21—H21C	0.9600	C15—H15A	0.9300
S3X—C21X	1.75 (4)	C16—C17	1.375 (8)
S3X—C16	1.75 (3)	C17—C18	1.393 (7)
C21X—H21D	0.9600	C17—H17A	0.9300
C21X—H21E	0.9600	C18—H18A	0.9300
C21X—H21F	0.9600	C19—H19A	0.9600
O1—C22	1.124 (6)	C19—H19B	0.9600
O2—C23	1.155 (6)	C19—H19C	0.9600
			
C23—Os1—C22	88.8 (2)	H21D—C21X—H21F	109.5
C23—Os1—C24	88.3 (2)	H21E—C21X—H21F	109.5
C22—Os1—C24	176.7 (2)	C2—C1—C6	118.8 (4)
C23—Os1—As1	102.89 (16)	C2—C1—As1	122.1 (3)
C22—Os1—As1	90.24 (14)	C6—C1—As1	119.1 (3)
C24—Os1—As1	88.87 (14)	C1—C2—C3	120.6 (4)
C23—Os1—Os3	97.90 (16)	C1—C2—H2A	119.7
C22—Os1—Os3	88.04 (14)	C3—C2—H2A	119.7
C24—Os1—Os3	93.91 (14)	C4—C3—C2	120.9 (4)
As1—Os1—Os3	159.100 (13)	C4—C3—H3A	119.5
C23—Os1—Os2	157.23 (16)	C2—C3—H3A	119.5
C22—Os1—Os2	94.70 (14)	C5—C4—C3	117.7 (4)
C24—Os1—Os2	88.54 (15)	C5—C4—S1	117.7 (4)
As1—Os1—Os2	99.586 (13)	C3—C4—S1	124.7 (4)
Os3—Os1—Os2	59.847 (8)	C4—C5—C6	121.9 (4)
C26—Os2—C28	101.9 (2)	C4—C5—H5A	119.1
C26—Os2—C27	89.2 (2)	C6—C5—H5A	119.1
C28—Os2—C27	90.9 (2)	C5—C6—C1	120.1 (4)
C26—Os2—C25	88.5 (3)	C5—C6—H6A	120.0
C28—Os2—C25	95.8 (2)	C1—C6—H6A	120.0
C27—Os2—C25	173.2 (2)	C12—C7—C8	118.3 (4)
C26—Os2—Os3	167.70 (16)	C12—C7—As1	119.5 (3)
C28—Os2—Os3	90.40 (17)	C8—C7—As1	121.9 (3)
C27—Os2—Os3	91.81 (16)	C9—C8—C7	120.8 (5)
C25—Os2—Os3	89.11 (19)	C9—C8—H8A	119.6
C26—Os2—Os1	108.61 (16)	C7—C8—H8A	119.6
C28—Os2—Os1	149.54 (17)	C10—C9—C8	120.6 (5)
C27—Os2—Os1	89.70 (16)	C10—C9—H9A	119.7
C25—Os2—Os1	85.01 (16)	C8—C9—H9A	119.7
Os3—Os2—Os1	59.148 (8)	C9—C10—C11	118.7 (5)
C30—Os3—C32	101.0 (3)	C9—C10—S2	130.7 (5)
C30—Os3—C31	91.4 (3)	C11—C10—S2	110.6 (5)
C32—Os3—C31	91.2 (2)	C9—C10—S2X	109.6 (5)
C30—Os3—C29	91.0 (4)	C11—C10—S2X	131.7 (6)
C32—Os3—C29	90.0 (3)	C12—C11—C10	120.3 (5)
C31—Os3—C29	177.1 (3)	C12—C11—H11A	119.9
C30—Os3—Os1	96.2 (2)	C10—C11—H11A	119.9
C32—Os3—Os1	162.78 (18)	C7—C12—C11	121.2 (5)
C31—Os3—Os1	86.62 (15)	C7—C12—H12A	119.4
C29—Os3—Os1	91.50 (18)	C11—C12—H12A	119.4
C30—Os3—Os2	157.2 (2)	C14—C13—C18	118.2 (4)
C32—Os3—Os2	101.86 (18)	C14—C13—As1	120.0 (3)
C31—Os3—Os2	87.73 (15)	C18—C13—As1	121.8 (3)
C29—Os3—Os2	89.4 (2)	C13—C14—C15	120.8 (5)
Os1—Os3—Os2	61.005 (6)	C13—C14—H14A	119.6
C13—As1—C7	101.94 (18)	C15—C14—H14A	119.6
C13—As1—C1	101.79 (18)	C14—C15—C16	120.9 (5)
C7—As1—C1	100.94 (18)	C14—C15—H15A	119.5
C13—As1—Os1	117.92 (12)	C16—C15—H15A	119.5
C7—As1—Os1	115.62 (13)	C17—C16—C15	119.0 (4)
C1—As1—Os1	116.07 (12)	C17—C16—S3X	117.6 (10)
C4—S1—C19	104.5 (3)	C15—C16—S3X	123.1 (10)
C10—S2—C20	99.4 (6)	C17—C16—S3	122.7 (7)
S2—C20—H20A	109.5	C15—C16—S3	118.2 (7)
S2—C20—H20B	109.5	C16—C17—C18	119.3 (5)
H20A—C20—H20B	109.5	C16—C17—H17A	120.3
S2—C20—H20C	109.5	C18—C17—H17A	120.3
H20A—C20—H20C	109.5	C13—C18—C17	121.7 (5)
H20B—C20—H20C	109.5	C13—C18—H18A	119.1
C20X—S2X—C10	99.1 (9)	C17—C18—H18A	119.1
S2X—C20X—H20D	109.5	S1—C19—H19A	109.5
S2X—C20X—H20E	109.5	S1—C19—H19B	109.5
H20D—C20X—H20E	109.5	H19A—C19—H19B	109.5
S2X—C20X—H20F	109.5	S1—C19—H19C	109.5
H20D—C20X—H20F	109.5	H19A—C19—H19C	109.5
H20E—C20X—H20F	109.5	H19B—C19—H19C	109.5
C16—S3—C21	104.6 (10)	O1—C22—Os1	175.3 (4)
S3—C21—H21A	109.5	O2—C23—Os1	177.4 (5)
S3—C21—H21B	109.5	O3—C24—Os1	174.4 (5)
H21A—C21—H21B	109.5	O4—C25—Os2	174.3 (6)
S3—C21—H21C	109.5	O5—C26—Os2	175.7 (5)
H21A—C21—H21C	109.5	O6—C27—Os2	175.0 (5)
H21B—C21—H21C	109.5	O7—C28—Os2	178.4 (5)
C21X—S3X—C16	101.8 (16)	O8—C29—Os3	177.2 (5)
S3X—C21X—H21D	109.5	O9—C30—Os3	176.4 (8)
S3X—C21X—H21E	109.5	O10—C31—Os3	174.9 (5)
H21D—C21X—H21E	109.5	O11—C32—Os3	177.9 (6)
S3X—C21X—H21F	109.5		
			
C6—C1—C2—C3	−0.3 (8)	C9—C10—C11—C12	−0.6 (8)
As1—C1—C2—C3	−179.5 (4)	S2—C10—C11—C12	−179.9 (5)
C1—C2—C3—C4	0.1 (9)	S2X—C10—C11—C12	175.7 (7)
C2—C3—C4—C5	0.0 (8)	C8—C7—C12—C11	2.9 (7)
C2—C3—C4—S1	179.5 (4)	As1—C7—C12—C11	−171.9 (4)
C19—S1—C4—C5	163.2 (5)	C10—C11—C12—C7	−2.2 (8)
C19—S1—C4—C3	−16.3 (6)	C18—C13—C14—C15	1.6 (8)
C3—C4—C5—C6	0.3 (8)	As1—C13—C14—C15	−177.4 (5)
S1—C4—C5—C6	−179.3 (4)	C13—C14—C15—C16	−2.0 (9)
C4—C5—C6—C1	−0.5 (8)	C14—C15—C16—C17	1.0 (9)
C2—C1—C6—C5	0.6 (8)	C14—C15—C16—S3X	−173.2 (14)
As1—C1—C6—C5	179.7 (4)	C14—C15—C16—S3	178.0 (9)
C12—C7—C8—C9	−0.9 (7)	C21X—S3X—C16—C17	163.8 (12)
As1—C7—C8—C9	173.8 (4)	C21X—S3X—C16—C15	−22 (2)
C7—C8—C9—C10	−1.9 (8)	C21—S3—C16—C17	4.3 (15)
C8—C9—C10—C11	2.6 (8)	C21—S3—C16—C15	−172.7 (8)
C8—C9—C10—S2	−178.3 (5)	C15—C16—C17—C18	0.3 (8)
C8—C9—C10—S2X	−174.5 (6)	S3X—C16—C17—C18	174.9 (13)
C20—S2—C10—C9	15.4 (10)	S3—C16—C17—C18	−176.6 (9)
C20—S2—C10—C11	−165.3 (8)	C14—C13—C18—C17	−0.3 (7)
C20X—S2X—C10—C9	161.1 (12)	As1—C13—C18—C17	178.7 (4)
C20X—S2X—C10—C11	−15.4 (15)	C16—C17—C18—C13	−0.7 (8)

### Undecacarbonyl{tris[4-(methylsulfanyl)phenyl]arsine}-triangulo-triosmium(0) Hydrogen-bond geometry (Å, º)

**Table:**

*D*—H···*A*	*D*—H	H···*A*	*D*···*A*	*D*—H···*A*
C5—H5*A*···O10^i^	0.93	2.55	3.41 (1)	154

Symmetry code: (i) −*x*+1, −*y*+2, −*z*+2.

## References

[bb1] Adams, C. J., Bruce, M. I., Duckworth, P. A., Humphrey, P. A., Kühl, O., Tiekink, E. R. T., Cullen, W. R., Braunstein, P., Coco Cea, S., Skelton, B. W. & White, A. H. (1994). *J. Organomet. Chem.* **467**, 251–281.

[bb2] Ang, H. G., Kwik, W. L., Leong, W. K. & Potenza, J. A. (1989). *Acta Cryst.* C**45**, 1713–1715. 10.1107/s0108270189003719 2610961

[bb3] Biradha, K., Hansen, V. M., Leong, W. K., Pomeroy, R. K. & Zaworotko, M. J. (2000). *J. Cluster Sci.* **11**, 285–306.

[bb4] Bruce, M. I., Liddell, M. J., Hughes, C. A., Patrick, J. M., Skelton, B. W. & White, A. H. (1988*b*). *J. Organomet. Chem.* **347**, 181–205.

[bb5] Bruce, M. I., Liddell, M. J., Hughes, C. A., Skelton, B. W. & White, A. H. (1988*a*). *J. Organomet. Chem.* **347**, 157–180.

[bb6] Bruker (2012). *APEX2* and *SAINT*. Bruker AXS Inc., Madison, Wisconsin, USA.

[bb7] Caracelli, I., Zukerman-Schpector, J., Haiduc, I. & Tiekink, E. R. T. (2016). *CrystEngComm* **18**, 6960–6978.

[bb8] Chen, Y., Zhen, Q., Meng, F.-J. Y. P., Yu, P. & Xu, C. (2024). *Chem. Rev.* **124**, 13370–13396. 10.1021/acs.chemrev.4c00516 39535080

[bb9] Groom, C. R., Bruno, I. J., Lightfoot, M. P. & Ward, S. C. (2016). *Acta Cryst.* B**72**, 171–179. 10.1107/S2052520616003954 PMC4822653 27048719

[bb10] Hansen, V. M., Ma, A. K., Biradha, K., Pomeroy, R. K. & Zaworotko, M. J. (1998). *Organometallics* **17**, 5267–5274.

[bb11] Honaker, M. T., Hovland, J. M. & Nicholas Salvatore, R. (2007). *Curr. Org. Synth.* **4**, 31–45.

[bb12] Krause, L., Herbst-Irmer, R., Sheldrick, G. M. & Stalke, D. (2015). *J. Appl. Cryst.* **48**, 3–10. 10.1107/S1600576714022985 PMC4453166 26089746

[bb13] Nicholls, J. N., Vargas, M. D., Deeming, A. J. & Kabir, S. E. (1990). *Inorg. Synth*. **28**, 232-235.

[bb14] Oh, S. P., Li, Y.-Z. & Leong, W. K. (2015). *J. Organomet. Chem.* **783**, 46–48.

[bb15] Raithby, P. R. (2024). *J. Organomet. Chem.* **1005**, 122979.

[bb16] Shawkataly, O. B., Khan, I. A., Sirat, S. S., Yeap, C. S. & Fun, H.-K. (2010). *Acta Cryst.* E**66**, m1047–m1048. 10.1107/S1600536810029223 PMC3007950 21588476

[bb17] Sheldrick, G. M. (2015*a*). *Acta Cryst.* A**71**, 3–8.

[bb18] Sheldrick, G. M. (2015*b*). *Acta Cryst.* C**71**, 3–8.

[bb19] Spackman, P. R., Turner, M. J., McKinnon, J. J., Wolff, S. K., Grimwood, D. J., Jayatilaka, D. & Spackman, M. A. (2021). *J. Appl. Cryst.* **54**, 1006–1011. 10.1107/S1600576721002910 PMC8202033 34188619

[bb21] Spek, A. L. (2020). *Acta Cryst.* E**76**, 1–11. 10.1107/S2056989019016244 PMC6944088 31921444

[bb22] Tan, S. L., Jotani, M. M. & Tiekink, E. R. T. (2019). *Acta Cryst.* E**75**, 308–318. 10.1107/S2056989019001129 PMC6399703 30867939

